# Targeting alternative splicing as a new cancer immunotherapy-phosphorylation of serine arginine-rich splicing factor (SRSF1) by SR protein kinase 1 (SRPK1) regulates alternative splicing of PD1 to generate a soluble antagonistic isoform that prevents T cell exhaustion

**DOI:** 10.1007/s00262-023-03534-z

**Published:** 2023-11-16

**Authors:** Mussarat Wahid, Benjamart Pratoomthai, Isioma U. Egbuniwe, Hannah R. Evans, Roya Babaei-Jadidi, Jason O. Amartey, Viola Erdelyi, Kiren Yacqub-Usman, Andrew M. Jackson, Jonathan C. Morris, Poulam M. Patel, David O. Bates

**Affiliations:** 1 Division of Cancer and Stem Cells, School of Medicine, Centre for Cancer Science, Biodiscovery Institute, University of Nottingham, Nottingham, NG2 7UH UK; 2 Department of Basic Medical Science, Faculty of Medicine Vajira Hospital, Navamindradhiraj University, 681 Samsen Road, Dusit, 10300 Bangkok Thailand; 3 School of Chemistry, University of New South Wales, Sydney, Australia; 4 COMPARE University of Birmingham and University of Nottingham, Midlands, UK

**Keywords:** RNA splicing, SRPK1, PD1, Immunotherapy

## Abstract

**Background:**

Regulation of alternative splicing is a new therapeutic approach in cancer. The programmed cell death receptor 1 (PD-1) is an immunoinhibitory receptor expressed on immune cells that binds to its ligands, PD-L1 and PD-L2 expressed by cancer cells forming a dominant immune checkpoint pathway in the tumour microenvironment. Targeting this pathway using blocking antibodies (nivolumab and pembrolizumab) is the mainstay of anti-cancer immunotherapies, restoring the function of exhausted T cells. PD-1 is alternatively spliced to form isoforms that are either transmembrane signalling receptors (flPD1) that mediate T cell death by binding to the ligand, PD-L1 or an alternatively spliced, soluble, variant that lacks the transmembrane domain.

**Methods:**

We used PCR and western blotting on primary peripheral blood mononuclear cells (PBMCs) and Jurkat T cells, IL-2 ELISA, flow cytometry, co-culture of melanoma and cholangiocarcinoma cells, and bioinformatics analysis and molecular cloning to examine the mechanism of splicing of PD1 and its consequence.

**Results:**

The soluble form of PD-1, generated by skipping exon 3 (∆Ex3PD1), was endogenously expressed in PBMCs and T cells and prevents cancer cell-mediated T cell repression. Multiple binding sites of SRSF1 are adjacent to PD-1 exon 3 splicing sites. Overexpression of phosphomimic SRSF1 resulted in preferential expression of flPD1. Inhibition of SRSF1 phosphorylation both by SRPK1 shRNA knockdown and by a selective inhibitor, SPHINX31, resulted in a switch in splicing to ∆Ex3PD1. Cholangiocarcinoma cell-mediated repression of T cell IL-2 expression was reversed by SPHINX31 (equivalent to pembrolizumab).

**Conclusions:**

These results indicate that switching of the splicing decision from flPD1 to ∆Ex3PD1 by targeting SRPK1 could represent a potential novel mechanism of immune checkpoint inhibition in cancer.

## Introduction

In cancers, widespread alteration in alternative splicing of multiple genes has been identified. In myeloid leukaemia evidence has been produced for a therapeutic approach targeting components of the spliceosome such as SF3B1 [1], and of controlling regulators of alternative splicing such as the SR protein kinase SRPK1 [2, 3]. These are moving into the clinic with phase I trials of SF3B1. However, these have been targets in the cancer cells themselves. The tumour microenvironment, and in particular the immune system, is a key regulator of tumour growth and metastasis, but also could be a target for alternative splicing therapy.

In many cancers tumour progression and metastasis occur because the immune system of the patient cannot target cancer cells. This is in part because the cancer cells develop the ability to control the host immune system by repressing immune cells that would normally target the cancer as being a foreign object – and in particular the T cell. There are multiple pathways through which cancer cells can repress the immune system, including validated targets that now form the bedrock of immunotherapy, including the PD1-PDL1 and the CTLA4-CD80/86 pathways. Programmed cell death ligand-1 (PD-L1), the target of atezolizumab, durvalumab and others acts on the programmed cell death receptor -1, (PDCD1 or PD-1), the target of nivolumab, pembrolizumab and others, on T cells and induces T cell exhaustion, preventing the T cell from mounting an immune response against the cancer. The cytotoxic T-lymphocyte-associated-4 (CTLA4) protein binds to CD80 and CD86 ligands on antigen presenting cells and prevents the stimulatory CD28 from activating T cells. CTLA4 is the target of ipilimumab, the first checkpoint inhibitor to be licensed, and tremelimumab. Alternative splicing of these molecules, particularly resulting in exclusion of the transmembrane domain, could lead to soluble endogenous inhibitors and such alternative splicing has been shown for CD28 [4], CD80 [5], CD86 [6], CTLA4 [7] and for PD-1 [8].

**Fig. 1:**
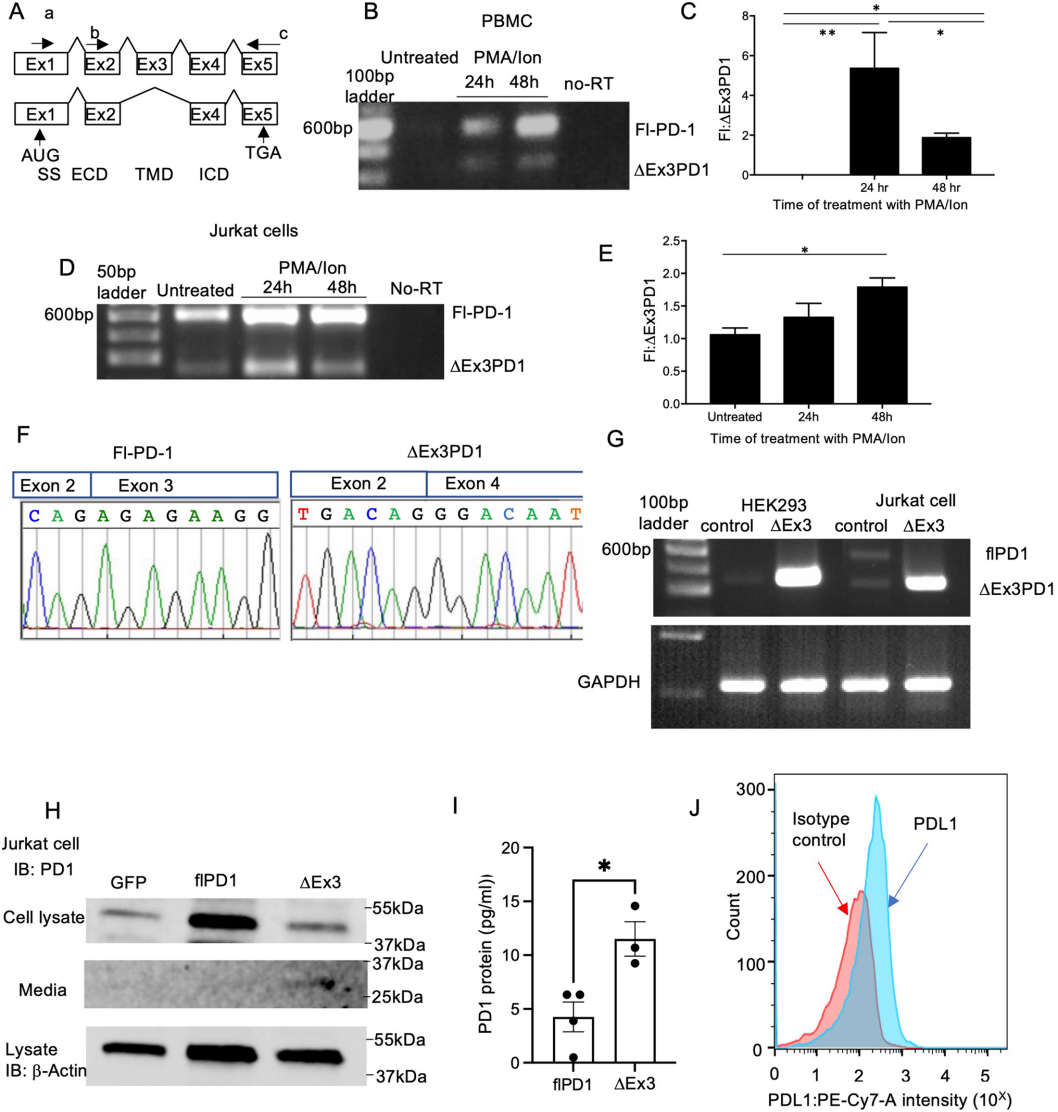
Antagonistic isoforms of PD-1 are produced by alternative splicing with and without activation. (**A**) Exon structure of PD-1. Primers used for determination of splicing isoforms shown by arrows a-primer used for cloning ∆Ex3PD1. b. Primer used for expression analysis. c. Reverse primer. Domains encoded by the sequence shown. SS = Signal sequence, ECD = extracellular (ligand-binding) domain, TMD = transmembrane domain, ICD = intracellular (signalling) domain). AUG = start site, TGA = stop codon. (**B**) PBMC isolated from healthy donors were treated with PMA and ionomycin at 24 h and 48 h and PCR performed for PD-1 using b and c primers (**C**) Quantification of intensity of flPD1 to ∆Ex3PD1. PBMC showed the expression of both PD-1 and ∆Ex3PD1 after the cells were activated with PMA and ionomycin. (**D**) PD-1 RNA expression in Jurkat T cells before and after treatment with PMA and ionomycin. (**E**) Quantification showed an increase in the expression of the ratio of flPD1 to ∆Ex3PD1 after activation. (**F**) Cloning of flPD1 and ∆Ex3PD1 from Jurkat cells confirmed by sequencing. (**G**) The cDNA encoding the coding sequence of the ∆Ex3PD1 variant was cloned into a pcDNA3 expression vector and transfected into HEK cells and Jurkat cells and RNA examined by RT-PCR. (**H**) Protein expression of flPD1 in cell lysate and secreted ∆Ex3PD1 in the media was confirmed by (**H**) western blotting and (**I**) ELISA (unpaired *t* test). (**J**) PD-L1 expression in MM418 melanoma cells was confirmed by flow cytometry after stimulation with IFN-γ. *p < 0.05, **p < 0.01

The *pdcd1* gene consists of 5 exons with each exon encoding different components of the protein; Exon 1 the signal sequence, Exons 1 and 2 the extracellular ligand binding domains, exon 3 the transmembrane domain and exons 4 and 5 the intracellular signalling domains. (Fig. 1A). The full-length functional PD-1 receptor (262 amino acids, 32 kDa, but with glycosylation, approximately 50 kDa) requires all five exons but alternative splicing of exon 3 (∆Ex3) could result in a protein lacking the 52 amino acids encoded by exon 3, which includes the entire transmembrane domain, and hence produce a soluble version of PD-1 protein (25 kDa) that would be secreted from the T cell, (∆Ex3PD1, a soluble PD-1, or sPD1) which could bind to PD-L1 and PD-L2, and prevent activation of the endogenous full-length receptor. This PD-1 splice variant was originally shown to be present by Nielsen et al. in 2015 [8] and has now been shown in coeliac disease [9]**,** but its role has not been explored, nor the control of splicing found. As a therapeutic strategy, switching the splicing from flPD1 to ∆Ex3PD1 could prevent cancer cell-mediated T cell exhaustion and enable an anti-cancer immune response. We therefore aimed to test the hypothesis that alternative splicing of PD-1 could result in checkpoint inhibition.

## Material and methods

### Cell culture, activation and IL-2 analysis by ELISA

Jurkat and MM418 cell lines were cultured in complete RPMI-1640 medium (Sigma-Aldrich, R0883) with 10% FBS (supplemented with 1% L-glutamine and 1% penicillin/streptomycin), and KKU-M055 cells were cultured in complete DMEM medium (Sigma-Aldrich, F7524) with 10% FBS as mention above, but without 1% penicillin/streptomycin. Additionally, complete DMEM medium was also used for Jurkat and KKU-M055 cells co-culture. Peripheral blood mononuclear cells (PBMCs) were isolated by density gradient centrifugation using a Ficoll-Paque solution (GE Healthcare) from heparinised peripheral blood samples. Isolated PBMCs were cultured in complete RPMI medium (supplemented with 10% FBS, 2 mM L-glutamine, 100 IU/ml penicillin and 100 µg/ml streptomycin and 0.1% 2-mercaptoethanol). Cells were cultured at 37 °C in a humidified atmosphere of 5% CO_2_ in air and transferred to fresh media every 3–4 days.

For IL-2 analysis, Jurkat cells were initially activated with 1 μg/ml ionomycin (Ion) and 10 nM phorbol myristate acetate (PMA) and the cell culture supernatant was analysed post 24 h and 48 h by IL-2 ELISA kit (BD OptEIA, #555,190) according to the manufacturer’s instructions. For co-culture experiment, MM418 cells were initially pre-treated with recombinant IFN-*γ* (1000 U/ml) for 48 h and were then harvested and cultured alone or co-cultured with Jurkat cells at different ratios. Jurkat cells were activated in the co-culture with PMA and ionomycin. Co-culture was incubated for 48 h and supernatants were collected and assessed for IL-2 expression by ELISA.

For Jurkat and KKU-M055 cells co-culture, the ratio was 1:6.4. KKU-M055 cells were activated with recombinant IFN-*γ* (1000 U/ml) for 48 h. Jurkat or ∆Ex3PD1-transfected Jurkat cells were activated with Ion and PMA as above for 24 h before cell co-culture. To test the effect of pembrolizumab on IL-2 production, activated Jurkat cells were co-cultured with activated KKU-M055 cells, and then pembrolizumab (Miltenyi Biotech, 130,095,293) added at various concentrations for another 48 h, the IgG-isotype was used as a control. For the testing of SPHINX31 effect on IL-2 production, Jurkat cells were treated with SPHINX31 at various concentrations and also activated with PMA and Ion as above for 24 h, DMSO was used as a control. Then the cells were co-cultured with activated KKU-M055 cells for another 48 h before observed under Leica phase contrast microscope. Cells were collected and analysed by qRT-PCR, and flow cytometry techniques.

For analysis of PD1 expression in media a soluble PD1 ELISA was undertaken according to the manufacturer’s instructions (Thermofisher BMS2214).

### Reverse-transcription PCR analysis

Total RNA was extracted from the cells by Nucleospin RNA kit (Macherey–nagel, # 740,984.50) according to the manufacturer's protocol. RNA (1 μg) was used to synthesise cDNA using first strand cDNA synthesis kit (Roche, # 11,483,188,001). PCR was performed using AmpliTaq Gold DNA Polymerase kit (Applied Biosystems, # 4,398,823) with thermocycling conditions: initial denaturation at 95 °C for 10 min, followed by 40 cycles of 95 °C for 30 s, 52 °C for 30 s, and 72 °C for 1 min; 1 cycle of melting curves, 72 °C for 10 min. PCR amplification products were run on 2% agarose gels (Bio-Rad). The mean intensity of bands was measured using Fiji [10].

### Quantitative RT-PCR (qRT-PCR)

Total RNA was extracted by using TRI reagent (Sigma-Aldrich, T9424) following the manufacturer’s instructions. Isolated RNA (1 µg) was used to synthesise complementary DNA using a PrimeScript RT reagent kit (Takara-Clontech, RR037A) following the manufacturer’s instructions. Quantitative RT-PCR (qRT-PCR) was performed by using SYBR Green master mix (Roche, 04707516001). The comparative CT method was applied for quantification of gene expression. GAPDH was used as endogenous controls. The following primers were used: IL-2, 5′-TACAACTGGAGCATTTACTG-3′ (forward) and 5′-GTTTCAGATCCCTTTAGTTC-3′ (reverse); GAPDH, 5′GAAGGTGAAGGTCGGAGTC-3′ (forward) and 5′-GAAGATGGTGATGGGATTTC-3′ (reverse).

### Flow cytometry analysis

Expression of human PD-L1 protein in stimulated (IFN-γ-1000 Unit/ml) and unstimulated MM418 cells was assessed by flow cytometry. Initially cells were activated with IFN-γ (1000 Unit/ml) for 48 h, 500,000 of the activated cells were resuspended in 1 ml of staining buffer (PBS-A, 1% FCS, 0.1% sodium azide). Cells were incubated with anti-PD-L1 antibody (B7-H1, PE-Cy (MIH1) Miltenyi Biotech) or an isotype control antibody for 30 min at 4 °C. To determine the survival rate of Jurkat cells after co-culture with KKU-M055 cells, 1 × 10^6^ cells were incubated with 1 µl of LIVE/DEAD Fixable Aqua Dead Cell Stain Kit (Invitrogen) in 1 ml of FACS buffer (PBS supplemented with 2% FBS) for 30 min at 4 °C. Cells were then washed once with FACS buffer and then incubated with anti-CD3 antibody (PerCP/Cy 5.5, Biolegend); or isotype control antibody for 30 min at 4 °C. Cells were then fixed with 4% formaldehyde and assessed for fluorescence. Data obtained were analysed by FlowJo or Kaluza software and graphs were generated by GraphPad Prism.

### Recombinant ∆Ex3PD1 plasmid construction

∆Ex3PD1 plasmid was generated by excising exon 3 from the flPD1 cDNA. Briefly, two sets of primers were designed to amplify exons 1 + 2 and exons 4 + 5. Primers used for the amplification of exon 1 + 2 were: forward 5’-AAGCTTCACCATGCAGATCCCACAGCGCCC-3’ and reverse 5’-CAATTGTCCCTGTCACCCTGAGCTCTGCC-3’. Primers used to amplify exon 4 + 5: forward 5’-CAATTGGAGCCAGGCGCACCGGCC-3’ and reverse 5’-ACTGGAAATCCAGCTCCCCA-3’. The two fragments was amplified individually and were then ligated and cloned into a pcDNA3 vector. Sequence of ∆Ex3PD1 was confirmed by sequencing.

### Minigene construction and mutation by site directed mutagenesis (SDM)

Minigene was constructed from the whole genomic sequence of PD-1. Briefly, PD-1 sequence containing introns were amplified by PCR using primers: forward 5’-ATGCAGATCCCACAGGCGCC-3’ and reverse 5’-TCAGAGGGGCCAAGAGCAGTG-3. The PD-1 amplified sequence was double digested with KpnI and BbVc1 restriction enzymes to excise intron 2. The remaining exons and introns were ligated into a pcDNA3 cloning vector. The final sequence was confirmed by sequencing and its expression was also analysed by RT-PCR after transfection into Jurkat cells.

Mutations were introduced in the minigene by SDM techniques. Initially, PCR primers were designed in which the specified bases were substituted in the middle of the sequence, with 18 non-overlapping nucleotides on each side. PCR mutagenesis was performed utilising the primers and the minigene plasmid using QuikChange XL II site directed mutagenesis kit (Agilent technologies) according to the manufacturer’s instructions. Briefly, a 50 μl PCR reaction was prepared containing 50 ng of the template, 1.25 ng of each primer, 200 μM dNTPs and 2.5 units of Pfu DNA polymerase. The PCR cycles were initiated at 95 °C for 1 min to denature the template DNA, followed by 18 amplification cycles. Each amplification cycle consisted of 95 °C for 50 s, 68 °C for 50 s and 68 °C for 1 min/kb and a final extension at 68 °C for 7 min. The PCR product was digested with 10 units of *Dpn*I enzyme at 37 °C for 1 h and then proceeded for transformation in E. coli strain XL-1 competent cells. Transformed cells were spread on LB Agar plate and were grown overnight at 37 °C. Colonies were selected the next day and miniprep was performed to purify the DNA. The fidelity of mutations was confirmed by sequencing.

### Transfection of Jurkat cell by electroporation

Jurkat cells grown to 80% confluency were pelleted by centrifugation. 700 μl of cell culture medium and 20 μg of the plasmid was added to the cells. The cell suspension was then transferred to electroporation cuvette (E6-0070, 4 mm, Gene flow) and electroporation was performed at 750 capacitance and 220 V in the Gene Pulser II, Bio Rad electroporation system. The transfected cells were transferred to pre-warmed cell culture medium in a T75 flask and were incubated at 37 °C. The transfection efficiency was evaluated after 24 h by RT-PCR.

### RNA immunoprecipitation (RIP)

RNA immunoprecipitation (RIP) was performed using the EZ-Magna RIP RNA-Binding Protein Immunoprecipitation Kit (Merck Millipore) following manufacturer’s instruction. Minigene-transfected Jurkat cells were lysed, RNA was extracted and stored in − 80 °C. Beads provided in the RIP kit were incubated with 5ug of anti-SRSF1 monoclonal antibody (anti-ASF-1 (SF2); Abcam) for 30 min at room temperature. RNA stored in − 80 °C was added to the SRSF1-beads and was incubated at 4 °C overnight. RNA was pulled down from the SRSF1-beads and was proceeded to RT-PCR analysis.

### shRNA-mediated knockdown by spinoculation

Jurkat cells were transduced with SRPK1 shRNA by spinoculation. Cells were initially pelleted by centrifugation and 1 × 10^5^ cells/ml were transferred into two sterile 15 ml conical tubes: one for non-targeting scrambled control (GE health Dharmacon, # s-005000–01) and one for targeted SRPK1 shRNA (GE health Dharmacon, # v3SVHS00-4655884). Lentiviral particle solution was added to Jurkat cells such that the final multiplicity of infection (MOI = 10) equalled 10 per tube. Jurkat cells were centrifuged at 800 × g for 50 min at 32 °C. Virus containing medium was aspirated and fresh medium was added to the cells and were incubated at 37 °C for three days. After three days, old media was replaced with fresh media and cells were kept under constant antibiotic selection. SRPK1 knockdown was assessed by RT-PCR using SRPK1 specific primers.

### In silico analysis of splicing

The exons of PD-1 were analysed by the online available “ESE finder” software (http://krainer01.cshl.edu/cgibin/tools/ESE3/esefinder.cgi?process=home) to identify the potential SRSFs binding sites to the exonic splicing enhancers (ESEs) and to predict where mutations would disrupt the expression of the gene. Briefly, sequence of PD-1 was copied from NCBI and then pasted in the input box in the ESE finder. A search was conducted for the heavily studied SRSFs; SRSF1, SRSF2, SRSF5 and SRSF6. The frequency of individual nucleotides at positions within the sequence was used to create a scoring system to identify how likely a sequence is to bind to an SR protein. After analysing the sequence, the binding sites were determined in each exon.

### Statistical analysis

Statistical analysis was carried out in GraphPad Prism. One-way or two-way analysis of variance was carried out where appropriate, unless otherwise stated and post hoc Holm–Sidak tests were used to determine statistical significance between groups. *P* < 0.05 was considered statistically significant.

## Results

PBMCs were isolated from healthy donors. Unstimulated cells did not show PD-1 expression by PCR, but treatment with 1.3 µM ionomycin (Ion) and 10 nM phorbol myristate acetate (PMA) – both T cell activators—resulted in expression of both flPD-1 and ∆Ex3PD1 (Fig. 1B), with flPD1 being expressed more highly (Fig. 1C). In a T cell line (Jurkat cells) isoforms expressing both the full length and the short form were detected, and this expression was increased after treatment with ionomycin and PMA (Fig. 1D) with the balance of isoforms maintaining predominance of flPD-1 after treatment (Fig. 1E). Cloning and sequencing of the PCR products from PBMC and Jurkat cells confirmed that these products were flPD-1 containing the exon 2/3 junction and sPD1 containing the exon 2/4 junction (∆Ex3PD1 Fig. 1F).

To investigate if ∆Ex3PD1 could be an inhibitor of the PD-1/PD-L1 pathway the short isoform of PD-1 was cloned into an expression vector and transfected into HEK cells or Jurkat cells. Figure 1G shows expression of ∆Ex3PD-1 mRNA in both cell types. In HEK cells, which did not express endogenous ∆Ex3PD1, transfection with recombinant ∆Ex3PD1 expression plasmid (∆Ex3) resulted in high expression of ∆Ex3PD1. In Jurkat cells, expressing both isoforms, transfection of ∆Ex3 resulted in high mRNA expression (Fig. 1G) of ∆Ex3PD-1. Jurkat cells were then transfected with either a control plasmid (GFP), one containing the full-length PD-1 (flPD1), or a plasmid containing the ∆Ex3PD1 cDNA. Immunoblotting showed enhanced expression of PD-1 in the flPD1 transfected cell lysate but enhanced expression of PD-1 in the media of the ∆Ex3PD1 transfected cells, indicating that ∆Ex3PD1 cDNA but not flPD1 resulted in enhanced secretion of soluble PD-1 (Fig. 1H). This was confirmed using an ELISA for soluble PD-1 of the media, where transfection with flPD1 did not show enhanced amounts of soluble PD-1 in the media, whereas transfection with ∆Ex3PD1 cDNA did enhance the amount of soluble PD-1 in the media (Fig. 1I).

**Fig. 2:**
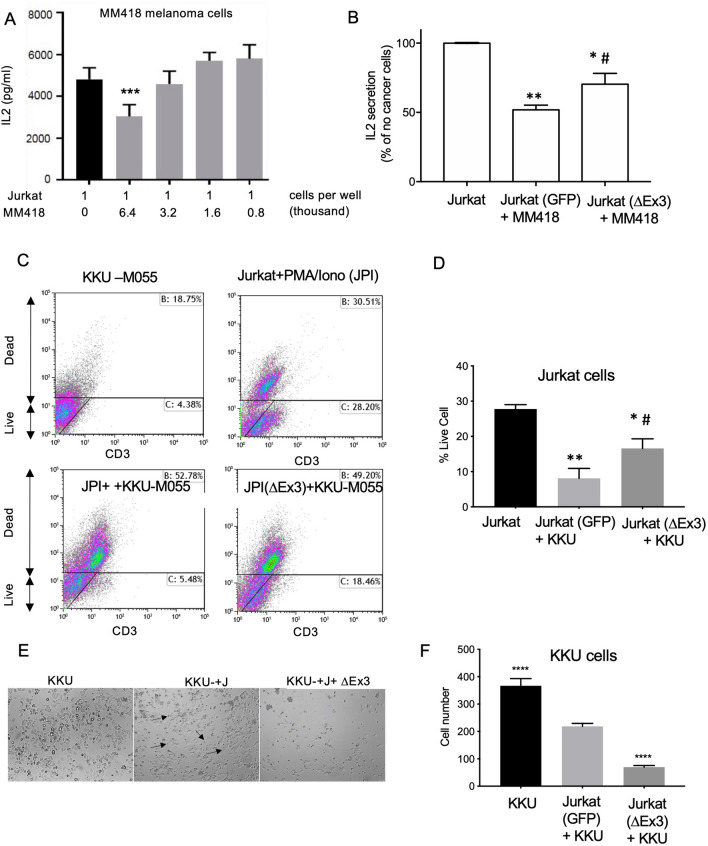
∆Ex3PD1 enhances T cell activation and survival and improves cancer cell killing. (**A**) Co-culture of Ion/PMA treated Jurkat cells (10,000 per well) with increasing number of IFN-γ treated MM418 cells showed inhibition of IL-2 production at a ratio of 6.4:1 cancer:T cells. (**B**) GFP transfected (control) or ∆Ex3PD1 transfected (∆Ex3) Jurkat cells were co-cultured with IFN-γ treated MM418 cells. The reduction in IL-2 production induced by melanoma cells was partially ameliorated by overexpression of ∆Ex3PD1 in Jurkat cells. (**C**) Flow cytometry of cells stained with live-dead stain and CD3 (T cells). (**D**) Transfection with ∆Ex3PD1 increased the proportion of live CD3 + cells when co-incubated with KKU-M055 cells. (**E**) Micrograph of KKU-M055 cancer cells alone, co-incubated with Jurkat cells (arrows) or Jurkat cells transfected with ∆Ex3PD1. (**F**) Co-culture of KKU-M055 cells with Jurkat cells transfected with GFP caused some KKU-M055 cell death, but over-expression of ∆Ex3PD1 significantly enhanced cancer cell death. *p < 0.05, **p < 0.01, ***p < 0.001, compared with Jurkat cells alone, ^#^*p* < 0.05 compared with GFP transfected cells

We then went on to determine whether this could inhibit T cell exhaustion induced by tumour cells. To determine whether ∆Ex3PD1 could reduce T cell exhaustion, we measured IL-2 mRNA in T cells expressing ∆Ex3PD1 after co-incubation of the T cells with IFN-γ-stimulated MM418 melanoma cells (expressing PD-L1, Fig. 1J). When seeded with Jurkat cells at a ratio of 6.4:1, co-incubation of Jurkat cells with these cells resulted in a significant inhibition of IL-2 production (Fig. 2A). Jurkat cells were then transfected with either a control vector expressing GFP or recombinant ∆Ex3PD1 plasmid (∆Ex3). When co-culturing the transfected Jurkat cells with MM418 cells the reduction in IL-2 production seen with the GFP transfected cells was significantly attenuated by transfection of ∆Ex3PD1 vector (Fig. 2B), indicating that ∆Ex3PD1 is capable of reducing T cell exhaustion by cancer cells. To determine whether ∆Ex3PD1 was able to enhance survival of T cells in the presence of cancer cells, we undertook flow cytometry with a live dead stain and CD3 staining for T cells in a Jurkat-cholangiocarcinoma co-culture (Fig. 2C). The proportion of live, activated (PMA and ionomycin treated) Jurkat cells was significantly decreased by the presence of the cholangiocarcinoma cell line KKU-M055 (Fig. 2D), and this was partially reversed when ∆Ex3PD1 was overexpressed. To determine whether ∆Ex3PD1 was able to enhance killing of cancer cells by T cells, KKU-M055 were co-incubated with activated Jurkat cells. This resulted in a visible killing of cholangiocarcinoma cells (Fig. 2E), which was enhanced by transfection of the Jurkat cells with the ∆Ex3 construct and confirmed by cell counts of the KKU-M055 (*p* < 0.001, Fig. 2F).

**Fig. 3:**
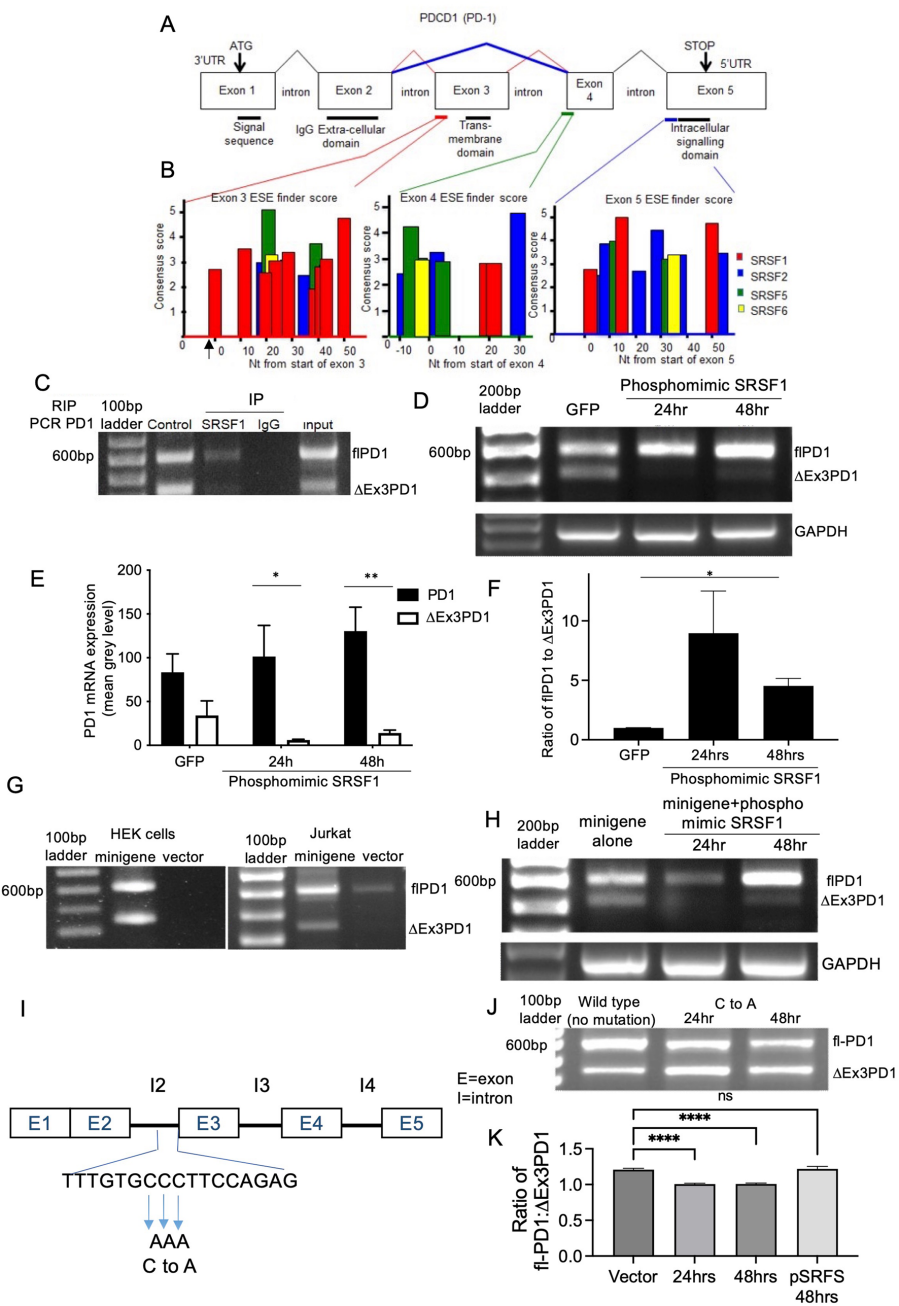
SRSF1 binds to and controls splicing of PD-1 in a phosphorylation-dependent manner. (**A**) Exon structure of the PD-1 gene. Sequences highlighted in red, green and blue were subjected to ESE finder analysis. Sequences encoding the functional domains are underlined. (**B**) Splice factor consensus sequences generated using ESE finder for the sequences around the splice acceptor sites for Exons 3, 4 and 5. (**C**) RNA immunoprecipitation showing SRSF1 pull down of flPD1. Jurkat cell lysate (control) was immunoprecipitated with anti-SRSF1 antibody or a control IgG immunoglobulin, or subjected to washing and exposure to beads without antibody (input). The RNA in the precipitate (or for the input the total RNA) was extracted and analysed by RT-PCR using PD-1 specific primers. (**D**) Jurkat cells were transfected with a plasmid expressing a phosphomimic, constitutively-active, nuclear-localised, SRSF1, and splicing of PD-1 measured by PCR 24 or 48 h after treatment with ionomycin and PMA. GFP – Jurkat cell transfected with GFP and treated for 24 h with ionomycin and PMA. (**E**) Quantification of PCR reactions from **D** flPD1 was increased and ∆Ex3PD1 decreased (N = 3). (**F**) Ratio of flPD1 to ∆Ex3PD1. (**G**) A minigene containing exons 1–2, intron 3, exon 3, intron 3, exon 4, intron 4 and exon 5 of PD-1 under control of the CMV promoter was transfected into HEK or Jurkat cells, and recapitulated endogenous splicing. (**H**) Splicing of the minigene was also switched to flPD1 by pSRSF1. (**I**) Mutations were generated in the sequence predicted by ESE finder in the polypyrimidine tract. (**J**) Expression of flPD1 and ∆Ex3PD1 after 24 (wild type minigene without mutation and mutated minigene) or 48 h (mutated minigene only) stimulation of Jurkat cells with PMA and ionomycin after mutation of polypyrimidine tract. (**K**) Quantitation of ratio of splice variants transfected with wild type minigene (vector), mutated minigene after 24 or 48 h, or co-transfection of the mutated minigene with the phosphomimic SRSF1 (pSRSF1, not shown in gel). *p < 0.05, **p < 0.01 compared with ∆Ex3PD1

To determine the likely control of PD-1 splicing we ran the sequences around the splice sites for exon 3, 4 and 5 (Fig. 3A) through ESE finder, a bioinformatic tool that predicts possible splice factor recognition sequences. It was striking that the exon 3 5’ splice site was highly enriched for SRSF1 sequences compared with exon 4 and 5 3’splice sites (Fig. 3B). To determine whether SRSF1 could bind to PD-1 mRNA we immunoprecipitated SRSF1 from Jurkat cells and subjected the precipitate to RNA extraction and RT-PCR, whereas both isoforms were present in control cells and in the input RNA flPD1 was only found in the SRSF1-immunoprecipitated RNA, and no ∆Ex3PD1 or flPD1 RNA in the IgG control-precipitated RNA (Fig. 3C) indicating that SRSF1 was preferentially associated with the full length over the short PD-1 isoform RNA. A constitutively active SRSF1 construct has previously been used to determine the role of SRSF1 in splicing [11]. A plasmid encoding this phosphomimetic SRSF1 was transfected into Jurkat cells and expression of PD-1 was examined. Whereas control vector transfected cells (GFP) had expression of both isoforms, ∆Ex3PD1 expression was not seen in the phosphomimetic transfected cells (Fig. 3D). Quantification of the isoforms indicated that there was an increase in flPD1 and reduction in ∆Ex3PD1 after transfection with phosphomimetic SRSF1 (Fig. 3E) resulting in an increase in the ratio of flPD1 to ∆Ex3PD1 (Fig. 3F). To be able to identify the sequences required for flPD1 expression, we generated a minigene containing the coding region of exon1 fused to exon 2, intron 2 (266nt), exon 3, intron 3 (119nt), exon 4, intron 4 (649nt) and the coding sequence of exon 5 under control of the CMV promoter. When transfected into HEK cells this resulted in transcription of both isoforms (Fig. 3G). When transfected into Jurkat cells this resulted in over-expression of both isoforms at a ratio that was similar to endogenous expression (Fig. 3G). When co-transfected with the phosphomimic SRSF1 this increased splicing to the flPD1 (Fig. 3H). To determine key splicing regulatory sequences required for the generation of flPD1 a C > A mutation of the three cytosines in the polypyrimidine tract, was made (Fig. 3I). The C > A mutation had a small effect on splicing of PD-1 (Fig. 3J), enhancing the production of the ∆Ex3 isoform (Fig. 3K), but the phosphomimic SRSF1 did not increase splicing to flPD1 in the mutant minigene as it did in the wild type minigene (Fig. 3H) or the endogenous gene as shown in Figs. 3D–F, suggesting that the C–A mutation prevents phosphorylated SRSF1 from substantively enhancing exon 3 inclusion. These results are consistent with phosphoSRSF1 binding to the intronic region upstream of the splice site and acting as an intronic splicing enhancer.

**Fig. 4:**
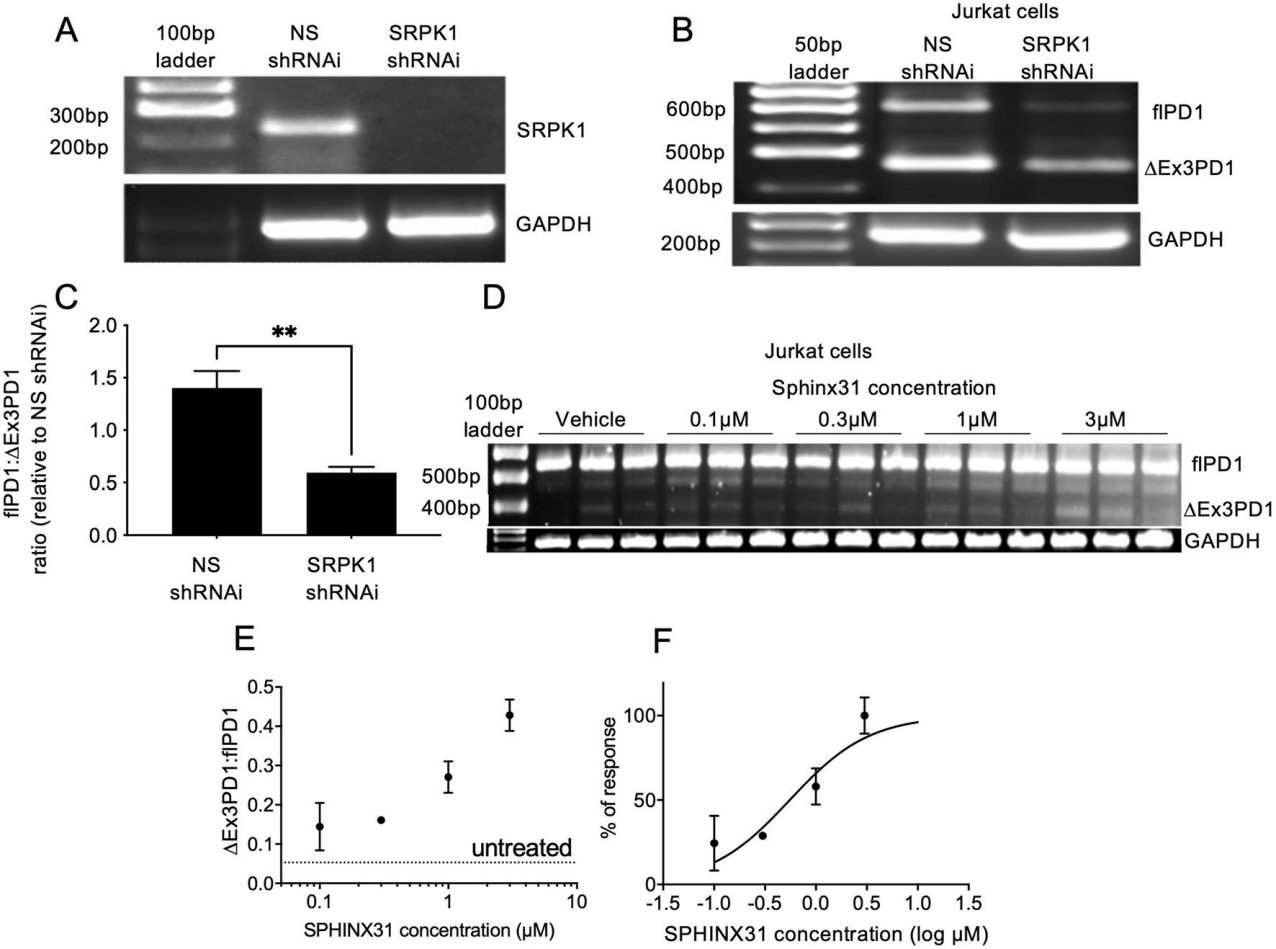
PD-1 splicing is controlled by SRPK1. (**A**) SRPK1 was knocked down by lentiviral transduction of shRNAi in Jurkat cells and expression measured by PCR. (**B**) PD-1 expression in SRPK1 knockdown cells was switched to ∆Ex3PD1. (**C**) Quantification of splicing shift in Jurkat cells after SRPK1 knockdown (unpaired *t* test). (**D**) Treatment of Jurkat cells with SRPK1 inhibitor SPHINX31 shows enhanced ∆Ex3PD1 expression. (**E**) Quantification of splicing shift demonstrates a dose-dependent increase. Dotted line shows baseline splicing level. (**F**) Curve fitting of normalised response demonstrates an IC50 of 550 nM. **p < 0.01

**Fig. 5:**
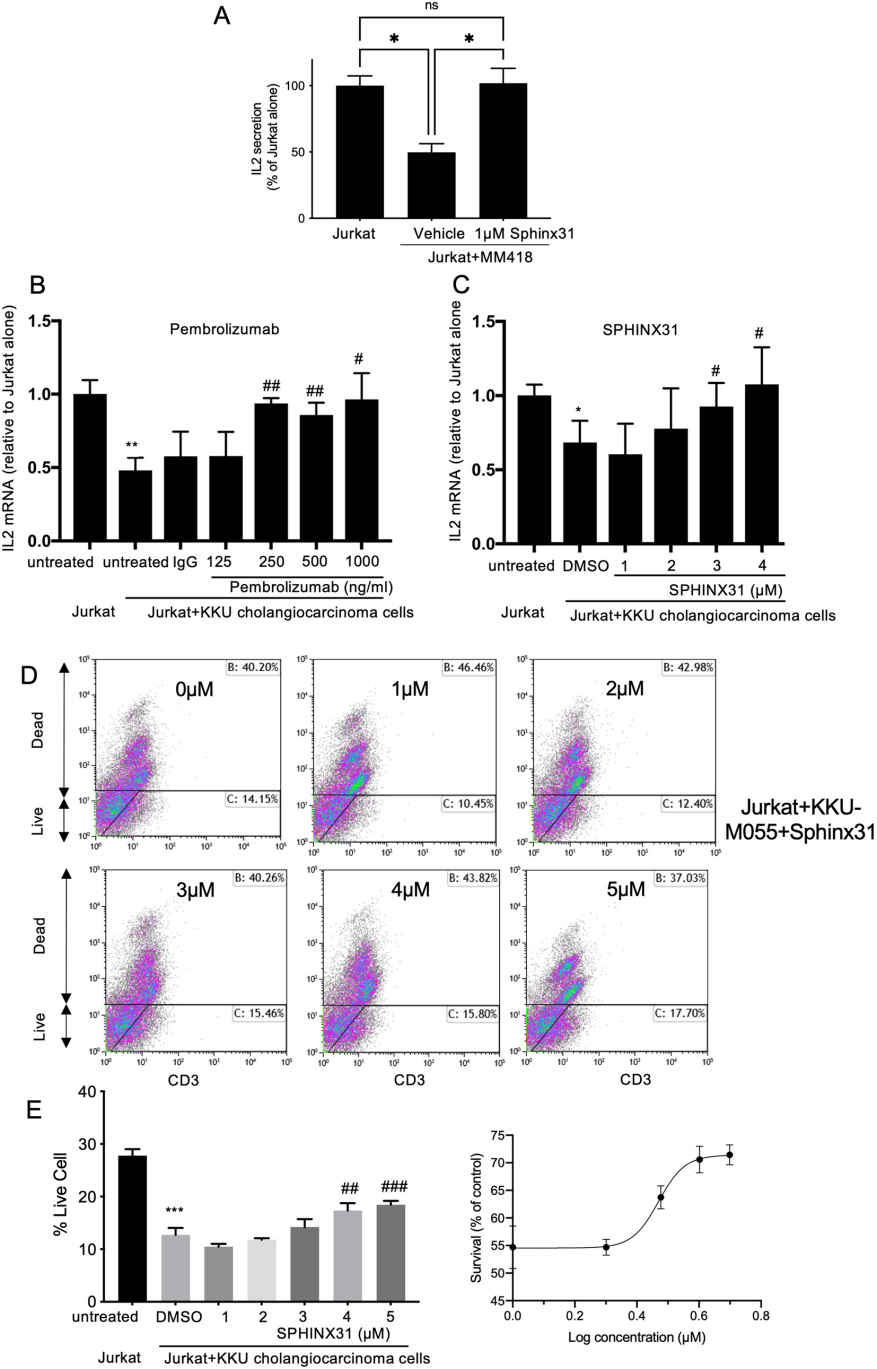
SRPK1 is a novel target for checkpoint inhibition (**A**) IL-2 ELISA of co-culture of Jurkat with MM418 melanoma cells in the presence or absence of SRPK1 inhibition. (**B**) IL-2 mRNA expression quantified by qRT-PCR in Jurkat co cultured with KKU-MO55 cells in the presence of increasing concentration of pembrolizumab (PD-1 antagonist) (N = 3). (**C**) SPHINX31 has an equivalent effect as pembrolizumab (N = 3). (**D**) Flow cytometry to measure live T cells (CD3 +) in Jurkat/KKUM055 cell co-culture. (**E**) SPHINX31 enhances T cell survival in co-culture with cancer cells. (**F**) Curve fitting of normalised response demonstrates an IC50 of 2.3 µM. *p < 0.05, ***p < 0.001 compared with untreated, ^#^*p* < 0.05, ^##^*p* < 0.01, ^###^*p* < 0.001 compared with vehicle control (IgG or DMSO)

SRSF1 is known to be phosphorylated by the splicing factor kinase SRPK1 [12]. We therefore investigated whether inhibition of SRPK1 could prevent splicing to flPD1 and result in a reversal of cancer cell-mediated T cell exhaustion. SRPK1 knockdown in Jurkat cells was achieved by lentiviral shRNAi transduction, resulting in a highly significant knockdown (Fig. 4A) and a switch in splicing from the flPD1 to ∆Ex3PD1 isoform (Fig. 4B, C). To determine whether this switch was amenable to pharmacological inhibition we used the SRPK1 selective inhibitor SPHINX31 [13]. Figure 4D shows that increasing concentrations of SPHINX31 resulted in increased ∆Ex3PD1 and a concentration-dependent increase in the ratio of ∆Ex3PD1:flPD1 (Fig. 4E). Nonlinear curve fitting to the normalised response showed an EC50 of 550 nM (95% CI 257–1065 nM, Fig. 4F). To determine whether this could result in inhibition of T cell exhaustion, Jurkat cells were co-cultured with MM418 melanoma cells in the presence or absence of pre-treatment with 1 µM SPHINX31 for 24 h. This resulted in a complete amelioration of the suppression of IL-2 production by activated Jurkat cells (Fig. 5A). To determine whether this was a generalised cancer response, we measured the effect on KKU-M055 cholangiocarcinoma cells and compared it with the effect of a PD-1 inhibitor pembrolizumab. KKU-M055 cells inhibited IL-2 mRNA expression in Jurkat cells when combined at a ratio of 6.4:1 cancer:T cells (Fig. 5B). This repression was dose dependently inhibited by pembrolizumab (Fig. 5B), and SPHINX31 (Fig. 5C). To determine whether this IL-2 response was sufficient to protect T cells, tumour cells and T cells were co-cultured with increasing concentrations of SPHINX31, after which cells were stained with live-dead and CD3 stains, and analysed by flow cytometry (Fig. 5D). Figure 5E shows that SPHINX31 partially reversed the reduction in live T cells induced by co-culture in a concentration-dependent manner with an EC50 of 2.7 µM (Fig. 5F).

## Discussion

Here we show that PD-1, the major target for immune checkpoint inhibition, has an endogenously expressed splice variant that acts as an endogenous inhibitor, and potentiates the immune response of T cells, and that this alternative splicing process is under control of the SR protein SRSF1 and can be switched by inhibition of its phosphorylation by SRPK1. Five different splice variants of PD-1 have been described, ∆Ex2,3,4, ∆Ex2,3 ∆Ex3 ∆Ex2 and flPD1, and the first three of these are likely to be soluble. However, only ∆Ex3 is likely to have the PD-L1 binding IgG domains. Our findings indicate that in stimulated T cells ∆Ex3 is a major isoform, although quantitatively the relative amounts remain to be determined, and RT-PCR can favour amplification of smaller isoforms, but it is clear that ∆Ex3 isoform can be a substantive component of the total PD-1 mRNA. Soluble PD-1 can be found circulating in plasma in both normal and cancer patients, but the origin of this circulating protein could come from either ectoprotein domain shedding (e.g. by proteolytic cleavage) [14] or alternative splicing. Blocking of PD-1/PD-L1 pathway using this ectodomain sPD1 has been shown to inhibit tumour growth and enhance immune response. The blockade of PD-L1 by this sPD1 also increases the transcription activities of IL-2 and IFN-γ genes [15]. Increased sPD1 levels in serum are associated with immune activation in chronic HBV infection suggesting sPD1 as a biomarker to immune activation and hepatocellular carcinoma development [16]. A study on patients with non-small cell lung cancer investigated the change in sPD1 concentration in the blood over the course of treatment with erlotinib, up until the development of clinical resistance, showed that patients with increased sPD1 levels during erlotinib treatment were associated with prolonged progression free survival and overall survival [17]. Another study on patients with cystic echinococcosis (CE, a chronic helminthic disease) showed an elevated concentration of sPD1 in the serum of CE patients as compared with the healthy control subjects [18]. The clinical relevance of sPD1 in rheumatoid arthritis (RA) patients is also indicated by significantly elevated plasma and synovial levels of sPD1, suggesting that it could be a useful marker for rheumatoid arthritis [19]. Similarly, elevated serum levels of sPD‐1 were observed in patients with systemic sclerosis and correlated with the extent of fibrosis and immunologic abnormalities [20]. However, current detection methodologies do not distinguish between proteolytically cleaved sPD1 and the ∆Ex3 splice variant, and the existing antibodies to sPD1 are targeted to the extracellular domain. However, in the ∆Ex3 splice variant the amino acids encoded by exons 4 and 5 are still present in the secreted protein, meaning that antibodies that specifically detect the exon 2-exon 4 splice junction could be generated to explore whether circulating sPD1 is from a splice or from a proteolytic cleavage event.

We showed here that exogenous expression of ∆Ex3PD1 was sufficient to partially reverse inhibition of IL-2 by melanoma and enhance killing of cholangiocarcinoma cells. Several studies have used recombinant soluble PD1 (rsPD1) to investigate the role of the extracellular domain of PD-1 in inhibiting ligand activation of the receptor. rsPD1 may functionally block the regulatory effect of flPD1 on T cells and may lead to alteration in T cell differentiation and regulation [21, 22]. rsPD1 can enhance T cell immunity [23] and rescue virus-specific CD4 and CD8 T cells proliferation during chronic infection [24, 25] and can facilitate anti-tumour immunity [15, 26]. This suggests a paracrine mechanism of action, although we have not shown that directly by using conditioned media, so these all support the concept that switching splicing to the ∆Ex3 splice variant could result in paracrine inhibition of PD-L1 and enhancement of T cell immunity.

Both the cancer cell types (melanoma [27] and cholangiocarcinoma [28]) used here have previously been shown to release PD-L1 to stimulate PD-1 and cause T cell exhaustion, but PD-L1 has also been shown to be released from tumour associated macrophages [29]. The mechanism of sPD1 action was assumed to be through binding to and preventing tumour cell PD-L1 from activating the endogenous PD-1 receptor, acting as a competitive inhibitor, but it could also function in vivo by preventing tumour associated macrophage-secreted PD-L1 or PD-L2. The reduction in IL-2 was noticeably less of an effect than the heightened killing of the tumour cells by the Jurkat cells. This could be due to synergy of activity between IL-2 and other cytotoxic cytokines (TNFa, IL-1, etc.), which we did not measure. Thus, the IL-2 output may be only a part of the cytotoxic effect enhanced by ∆Ex3PD1.

We then went on to show that PD-1 splicing was under control of SRSF1 binding and phosphorylation. SRSF1 has been proposed to act as either an exon splicing enhancer or intronic splicing repressor. The region of the RNA that mapped to SRSF1 was the start of the exon, and expression of phosphomimetic SRSF1 resulted in preferential inclusion of exon 3, indicating that SRSF1 was acting as an intronic splicing enhancer by binding in that region and so preventing exon skipping. It also indicates that the exon skip was enhanced by the mutation in the intron, again suggesting that in this circumstance SRSF1 might be acting as an intronic splicing enhancer. We did not investigate whether other RNA binding proteins could also interact, and this would need to be the subject of further study, so while it is clear that SRSF1 is involved, we cannot say that it is the only SR protein (or RNA binding protein) involved in splicing of PD-1.

Finally, we showed that control of SRSF1 phosphorylation by the kinase SRPK1 was able to enhance the splicing of the ∆Ex3 isoform. This is particularly interesting because it demonstrates that the balance of isoforms can be controlled. This contrasts with the results shown in Fig. 1 where exogenous ∆Ex3PD1 was able to slightly ameliorate the reduction in IL-2 production by Jurkat cells, as switching endogenous splicing reduces the amount of full-length PD-1 expressed by the same amount as increasing the ∆Ex3PD1 expressed, thus having a synergistic effect on inhibition of PD-L1 –there is more inhibitor and less receptor. Thus switching alternative splicing offers a potentially more attractive therapeutic approach than using monoclonal antibodies that can only act on the receptor, as it makes the T cell less able to respond, and produces its own endogenous inhibitor that can prevent PD-L1 from acting on other T cells as well. The range through which this can act has not yet been determined – by for instance investigating the effect of bulk media from ΔEx3PD1 expressing cells on co-cultured flPD1 expressing T cells with PDL1 expressing cancer cells.

It remains to be seen whether SRPK1 inhibitors or knockdown could be used in *in vivo* models—∆Ex3PD1 mRNA has not yet been described in mice, so more complex models might be necessary. The more muted effect of SRPK1 inhibition compared with knockdown may be due to drug efficacy, effect of protein expression rather than activation, or off target effects. (Although SPHINX31 is highly selective for SRPK1, it may also affect phosphorylation of other SR proteins.) Interestingly, SRPK1 inhibition also increased expression of a band between ∆Ex3PD1 and flPD1. This product has not been identified but could be another splice variant of PD-1. Although we have not shown here direct inhibition of SRSF1 phosphorylation by SPHINX31 in T cells, the concentrations of SPHINX31 used are consistent with previous studies showing inhibition of SRSF1 phosphorylation in epithelial cells [13]. We have also not shown directly that the effect of SRPK1 inhibition is a direct consequence of the switch in splicing of PD-1, for instance by showing that it is blocked by excess soluble PD-L1, but given the change in splicing of PD-1 induced by SRPK1 inhibition shown here, and the effect of the ∆Ex3PD1 isoform, the evidence points to this acting through a PD-1-mediated splicing pathway. The results shown here therefore suggest that SRPK1 could be a target in T cells in which PD-1 expression contributes to immune suppression in cancers.

## Conclusions

Immune checkpoint inhibitors are a key breakthrough in cancer therapy and are currently used as antibodies inhibitnfi PD-1 or its ligand PD-L1. We show here that PD1 is alternatively spliced to form an antagonistic, soluble version ∆Ex3PD1 in human T cells, which is under control of the splicing factor SRSF1 and its phosphorylation by SRPK1. These results go on to demonstrate that SRPK1 inhibition switches splicing of PD-1 to generate the antagonistic isoform, which enhances T cell killing of tumour cells, opening up the possibility of small molecule SRPK1 inhibitors as novel pharmacological immunotherapies.

## Data Availability

The datasets during and/or analysed during the current study available from the corresponding author on reasonable request.

## Abbreviations

CI: Confidence interval
CD: Cluster of differentiation
CMV: Cytomegalovirus
ΔEx3PD1: PD-1 generated by skipping exon 3
EC50: Excitation concentration 50%
ELISA: Enzyme-linked immunosorbent assay
ESE: Exon splicing enhancer
flPD1: Full-length PD-1
GFP: Green fluorescent protein
HBV: Hepatitis B virus
HEK: Human embryonic kidney
IFN-γ: Interferon gamma
IL-2: Interleukin-2
mRNA: Messenger RNA
PBMCs: Peripheral blood mononuclear cells
PCR: Polymerase chain reaction
PD-1, PCDR1: Programmed cell death receptor 1
PD-L1: PD-1 ligand 1
PMA: Phorbol myristol ester
rsPD1: Recombinant sPD1
SF3B1: Splicing factor 3B1
shRNAi: Short hairpin ribonucleic acid interference
sPD-1: Soluble PD-1
SRPK1: SR protein kinase 1
SRSF1: Serine arginine-rich splicing factor
TNFa: Tumour necrosis factor

## References

[1] Lee SC, North K, Kim E (2018). Synthetic lethal and convergent biological effects of cancer-associated spliceosomal gene mutations. Cancer Cell..

[2] Tzelepis K, De Braekeleer E, Aspris D (2018). SRPK1 maintains acute myeloid leukemia through effects on isoform usage of epigenetic regulators including BRD4. Nat Commun.

[3] Tzelepis K, Koike-Yusa H, De Braekeleer E (2016). A CRISPR dropout screen identifies genetic vulnerabilities and therapeutic targets in acute myeloid leukemia. Cell Rep.

[4] Magistrelli G, Jeannin P, Elson G, Gauchat JF, Nguyen TN, Bonnefoy JY, Delneste Y (1999). Identification of three alternatively spliced variants of human CD28 mRNA. Biochem Biophys Res Commun.

[5] Kakoulidou M, Giscombe R, Zhao X, Lefvert AK, Wang X (2007). Human Soluble CD80 is generated by alternative splicing, and recombinant soluble CD80 binds to CD28 and CD152 influencing T-cell activation. Scand J Immunol.

[6] Jeannin P, Magistrelli G, Aubry JP (2000). Soluble CD86 is a costimulatory molecule for human T lymphocytes. Immunity.

[7] Magistrelli G, Jeannin P, Herbault N, Benoit De Coignac A, Gauchat JF, Bonnefoy JY, Delneste Y (1999). A soluble form of CTLA-4 generated by alternative splicing is expressed by nonstimulated human T cells. Eur J Immunol.

[8] Nielsen C, Ohm-Laursen L, Barington T, Husby S, Lillevang ST (2005). Alternative splice variants of the human PD-1 gene. Cell Immunol.

[9] Ponce de Leon C, Lorite P, Lopez-Casado MA, Barro F, Palomeque T, Torres MI (2021). Significance of PD1 alternative splicing in celiac disease as a novel source for diagnostic and therapeutic target. Front Immunol.

[10] Schindelin J, Arganda-Carreras I, Frise E (2012). Fiji: an open-source platform for biological-image analysis. Nat Methods.

[11] Amin EM, Oltean S, Hua J (2011). WT1 mutants reveal SRPK1 to be a downstream angiogenesis target by altering VEGF splicing. Cancer Cell.

[12] Ghosh G, Adams JA (2011). Phosphorylation mechanism and structure of serine-arginine protein kinases. FEBS J.

[13] Batson J, Toop HD, Redondo C (2017). Development of potent, selective SRPK1 inhibitors as potential topical therapeutics for neovascular eye disease. ACS Chem Biol.

[14] Zhu X, Lang J (2017). Soluble PD-1 and PD-L1: predictive and prognostic significance in cancer. Oncotarget.

[15] Xiao H, Huang B, Yuan Y (2007). Soluble PD-1 facilitates 4–1BBL-triggered antitumor immunity against murine H22 hepatocarcinoma in vivo. Clin Cancer Res.

[16] Li N, Zhou Z, Li F, Sang J, Han Q, Lv Y, Zhao W, Li C, Liu Z (2017). Circulating soluble programmed death-1 levels may differentiate immune-tolerant phase from other phases and hepatocellular carcinoma from other clinical diseases in chronic hepatitis B virus infection. Oncotarget.

[17] Sorensen SF, Demuth C, Weber B, Sorensen BS, Meldgaard P (2016). Increase in soluble PD-1 is associated with prolonged survival in patients with advanced EGFR-mutated non-small cell lung cancer treated with erlotinib. Lung Cancer.

[18] Li Y, Xiao Y, Su M, Zhang R, Ding J, Hao X, Ma Y (2016). Role of soluble programmed death-1 (sPD-1) and sPD-ligand 1 in patients with cystic echinococcosis. Exp Ther Med.

[19] Greisen SR, Rasmussen TK, Stengaard-Pedersen K, Hetland ML, Horslev-Petersen K, Hvid M, Deleuran B (2014). Increased soluble programmed death-1 (sPD-1) is associated with disease activity and radiographic progression in early rheumatoid arthritis. Scand J Rheumatol.

[20] Fukasawa T, Yoshizaki A, Ebata S (2017). Contribution of soluble forms of programmed death 1 and programmed death ligand 2 to disease severity and progression in systemic sclerosis. Arthritis Rheumatol.

[21] Wan B, Nie H, Liu A, Feng G, He D, Xu R, Zhang Q, Dong C, Zhang JZ (2006). Aberrant regulation of synovial T cell activation by soluble costimulatory molecules in rheumatoid arthritis. J Immunol.

[22] Wu H, Miao M, Zhang G, Hu Y, Ming Z, Zhang X (2009). Soluble PD-1 is associated with aberrant regulation of T cells activation in aplastic anemia. Immunol Invest.

[23] Song MY, Park SH, Nam HJ, Choi DH, Sung YC (2011). Enhancement of vaccine-induced primary and memory CD8(+) T-cell responses by soluble PD-1. J Immunother.

[24] Onlamoon N, Rogers K, Mayne AE, Pattanapanyasat K, Mori K, Villinger F, Ansari AA (2008). Soluble PD-1 rescues the proliferative response of simian immunodeficiency virus-specific CD4 and CD8 T cells during chronic infection. Immunology.

[25] Amancha PK, Hong JJ, Rogers K, Ansari AA, Villinger F (2013). In vivo blockade of the programmed cell death-1 pathway using soluble recombinant PD-1-Fc enhances CD4+ and CD8+ T cell responses but has limited clinical benefit. J Immunol.

[26] Shin SP, Seo HH, Shin JH (2013). Adenovirus expressing both thymidine kinase and soluble PD1 enhances antitumor immunity by strengthening CD8 T-cell response. Mol Ther.

[27] Yang W, Chen PW, Li H, Alizadeh H, Niederkorn JY (2008). PD-L1: PD-1 interaction contributes to the functional suppression of T-cell responses to human uveal melanoma cells in vitro. Invest Ophthalmol Vis Sci.

[28] Liu Z, Li S, Zeng J (2020). LKB1 inhibits intrahepatic cholangiocarcinoma by repressing the transcriptional activity of the immune checkpoint PD-L1. Life Sci.

[29] Loeuillard E, Yang J, Buckarma E (2020). Targeting tumor-associated macrophages and granulocytic-myeloid-derived suppressor cells augments pd-1 blockade in cholangiocarcinoma. J Clin Invest.

